# Parents’ and nurses’ ideal collaboration in treatment-centered and home-like care of hospitalized preschool children – a qualitative study

**DOI:** 10.1186/s12912-020-00445-7

**Published:** 2020-06-09

**Authors:** Hildegunn Sundal, Solfrid Vatne

**Affiliations:** Molde University College Specialized University in Logistics, Faculty of Health Sciences and Social Care, Molde, Norway

**Keywords:** Children’s hospitalization, Preschool, Nurse-parent collaboration, Qualitative design, Field study, Interview

## Abstract

**Background:**

The hospitalization of children requires collaboration between parents and nurses in partnerships. This study examines parents’ and nurses’ experiences of ideal collaboration in treatment-centered and home-like care of hospitalized preschool children.

**Methods:**

This qualitative study is part of a larger study of 12 parents and 17 nurses who were responsible for 11 hospitalized children. Data collection took place at a Norwegian general paediatric unit, and the data were gathered from observations of and qualitative interviews with the parents and nurses. The analysis was conducted in six steps, in alignment with Braun and Clarke.

**Results:**

Two essential themes emerged from the analysis. (1) Treatment-centered care focuses on the following tasks in building relationships – gaining trust, securing – gaining voluntariness, distracting and comforting, and securing and gaining voluntariness. The purpose of treatment-centered care is to perform diagnostic procedures and offer treatment. (2) Home-like care, the purpose of which is to manage a child’s everyday situations in an unfamiliar environment, focuses on the following tasks: making familiar meals, maintaining normal sleeping patterns, adjusting to washing and dressing in new situations, and normalizing the time in between. From this pattern, we chose two narratives that capture the essence of ideal collaboration between parents and nurses.

**Conclusion:**

The ideal collaboration between nurses and parents is characterized by flexibility and reciprocity, and is based on verbal and action dialogues. In treatment-centered care, parent-nurse collaboration was successful in its flow and dynamic, securing the children’s best interests. Meanwhile, the achievement of the children’s best interest within home-like care varied according to the level of collaboration, which in turn was related to the complexity of the children’s everyday situations.

## Background

This article focuses on the collaboration between parents and nurses when preschool children are hospitalized. Evidence supports that creating a partnership with parents or other family members improves the quality of care for children with long-term illnesses, as well as the quality of paediatric nursing practice [1]. The philosophy behind this is that the family is the constant in the child’s life, and family-centered care offers a way of involving parents in the child’s care through partnership. However, actively involving parents in partnerships appears to be challenging. Smith, Swallow & Coyne [2], in a concept synthesis based on thirty studies, indicates the importance of supporting parents in their role, valuing parents’ knowledge and experiences, and incorporating parents’ expertise in developing effective parent-professional relationships as collaborative processes. However, the synthesis also suggests that implementation of these concepts into practice remains problematic, because of poor information sharing, lack of understanding of the family context, and not valuing parents’ knowledge and contribution [2–6].

Past studies on collaboration between nurses and parents have focused on collaboration between nurses and parents in relation to the performance of specific procedures and the treatment of hospitalized children. When the nurses/health personnel have the initiative and responsibility of organizing and performing the necessary tasks [7–13], the parents participated and assisted in procedural situations [10, 13], and their presence was considered important [7]. A number of studies have described the parents’ performance of daily basic care of hospitalized children. Parents mainly regard the following as their responsibility: for example, bathing and dressing the child, administering food, mobilizing the child, and comforting the child [7, 9, 10, 13–19]. In order to take care of the children, the parents needed the support and facilitation skills of the nurses [20]. This corresponds to the fact that nurses, due to their other duties, were unable to provide basic care for the children [10]. Aein et al., [8] emphasized that nurses and parents experience that they each have their specific domain in caring for the children. The way in which nurses and parents share the responsibility of caring for the child is in continuous flux [15].

The overall aim of this study is to explore the experiences of parents and nurses and the concrete ways in which nurses and parents collaborate in partnership when caring for hospitalized preschool children. From observation and responses of nurses and parents, the study explored the question of what is the ideal collaboration to fill the need of effective partnership in family-centered care.

## Methods

The study has a qualitative design based on a hermeneutical perspective where the basis is the understanding and interpretation of parents’ and nurses’ experiences in their everyday life. According to Gadamer, our understanding is influenced of our prejudices and the present must be understood in the light of the past. Our understanding of an experience is therefore always the fusion of both present and past based on a polarity of familiarity and strangeness [21]. To obtain a deeper understanding of the experiences of both nurses and parents, a field study with observations and interviews were performed [22, 23]. The analysis of the observations and interviews was based on a hermeneutical perspective, which guided interpretation of the parents’ and nurses’ actions and experiences [21].

### Participants

The study took place at a general medical paediatric unit in a Norwegian hospital. The criteria for selection were that the parents stayed with the child, the child was in the beginning stages of hospitalization, probably staying for 2 days or more, the child was neither critically nor terminally ill, the child was between one and 6 years old – preschool age in Norway; and the parents had Norwegian as their first language. We planned to include 10–15 preschool children with their respective parents and nurses, while leaving room to potentially expand or reduce the number of participants. The participating nurses had to be responsible for the selected children. To recruit participants, the head nurse directly asked parents to participate in the study, and invited individual nurses to participate, by sending an email, which instructed nurses to submit their answers in a locked box.

Twelve parents (three fathers and nine mothers) of 11 hospitalized children, and 17 female nurses participated in the observation (all were registered nurses [RN]; one nurse was also a paediatric nurse: their experiences in paediatric units ranged from 1 to 17 years). When 11 children were included in the population, less nuances and variations of the collaboration between parents and nurses was apparent in the sample. All parents and 13 nurses were interviewed during the observation period. For practical reasons, we did not interview all nurses included in the study, but rather gave priority to nurses who were responsible for the children on the day the children arrived and the day they left the unit. Six of the children’s hospitalizations were planned in advance, and five children were admitted with acute medical conditions. They had various medical diagnoses: four children had chronic medical disorders from birth. The children, eight girls and three boys, were hospitalized from two to 4 days, and one child was readmitted for 1 day. The children’s ages were between one and six as follows: two children were 1 year old, four were two, four were three, and one was six.

### Ethical considerations

Regional Committees for Medical and Health Research Ethics of Norway (4.2006.3865/4.2007.1097) and the Ministry of Health and Care Services of Norway (07/3088–14.06.2007) have approved this study. This study has been reported to the Norwegian Centre for Research Data (04.06.2007–16,697/JE). The head nurse of the children’s unit, who also obtained informed written consent from the parents, contacted the informants. We obtained informed written consent from the nurses before the start of the study. The participants were informed of their rights to confidentiality and voluntariness, how to participate in the study, and their right to withdraw at any time. Children are vulnerable due to their immaturity, and parents become vulnerable when their child suffers in an unfamiliar environment. This was addressed by spending time introducing the researcher (first author) both to the child and the parent(s) and by spending time for the researcher and participants to become acquainted, starting with small talk, before taking a more passive role. The researcher (first author) was conscious of her own preunderstanding and attempted to approach the participants with openness [23].

### Data collection

The primary researcher (first author) performed the data collection for this study over a period of 4 months, observing one child per week in an unstructured manner [22]. The researcher’s role was as a partial participant observer. That is, at the beginning of each observation period, the research stayed close to the situation, including participating in small talk, and then stepped back in the room in order not to affect the participants’ collaboration. The researcher sometimes participated, for example by giving equipment to a nurse. The researcher followed the nurse who was responsible for each child during every morning shift and some afternoon shifts until that child was discharged (27 morning shifts, 5 afternoon shifts – about 160 h). If procedures was planned for in the afternoon, the observation continued. Descriptive and reflective field notes were written retrospectively, shortly after the observed situations and often in the afternoon. The descriptions focused on personal relationships and movements, conversation, play, and the performance of practical tasks, including procedural situations. To supply the field notes, the first author conducted qualitative interviews with parents after the observation and with nurses at the time of the child’s discharge. The interviews and observations focused on the collaborative situations of parents and nurses, more precisely, on the participants’ actions and experiences of collaboration related to medical procedures, the child’s treatment, and topics such as the child’s sleep and meals. The observation and interview guide was thematically oriented with the themes: washing and dressing, meals and eating, sleeping, relief (i.e., the parents’ need to leave the child), play/activity, illness experiences (e.g., disease symptoms, discomfort and pain), and procedures and treatments. The aim of the interviews was to generate rich insights on the parents’ and nurses’ experiences of the observed collaborative situations. The interviews were performed in the hospital (except with one parent who was contacted via phone and one interviewed at home) and lasted from 30 to 90 min. The interviews were audio taped and transcribed verbatim. Field notes were written after each observation. The children were also assigned fictitious names to preserve anonymity, both in the field notes and the transcript.

### Data analysis

We organized the collected data thematically, based on actions and experiences. To do so, we applied the hermeneutic method, alternating between observed and interview details in a holistic approach [21]. The data from the observations provided important details for the analysis of the participants’ actions and events in collaboration situations, including the context. Meanwhile, the data from the interviews was important in analyzing parents’ and nurses’ understanding of the situations, their reactions, and the reasons why they acted in specific ways. We conducted a thematic analysis [24], which is a ‘bottom up’ and inductive way to identify themes and patterns in the data. The thematic analysis searches for patterns in the data, and the themes identified are strongly linked to the data itself [25]. During the analysis, we systematically examined the data to identify repeated patterns of meaning. This process was composed of six steps [24]. The first step, ‘familiarizing yourself with the data’, involved reading the entire transcript of material. We read the text several times to become familiar with it and, at the same time, to note ideas to encode – latent themes. The second step involved generating initial codes by identifying interesting aspects based on patterns, themes and notes in the text related to collaboration between parents and nurses. The preliminary themes developed were ‘everyday situations’ and ‘procedural situations’. In the third phase, we sorted the different codes into potential themes based on the discovered patterns. In the fourth step, we collated all the relevant coded data extracted into broader themes searching for relationships between themes and between different levels of themes and subthemes, thus making an overview. In the fifth step, we named the themes; the names captured something important about the data in relation to the research question: ‘treatment-centered care’ and ‘home-like care’. ‘Treatment-centered care’ included the following subthemes: 1) building relationships – gaining trust, 2) securing – gaining voluntariness, 3) distracting and comforting, and securing and gaining voluntariness. The second theme was ‘home-like care’ and included the following subthemes: 1) making familiar meals, 2) maintaining normal sleeping patterns, 3) adjusting washing and getting dressed in new situations, 4) normalizing the time in between (cf. Table 1). Then, for each individual theme, we wrote a detailed analysis and identified narratives in relation to the research question [24, 26].

**Table 1:** Examples of themes and subthemes

Meaning units from interview text and field notes/codes	Name of meaning units/codes	Themes	Subthemes
The nurse first greets the child and then the father, and involves herself in play with the child. When the nurse has played a short while with the child, she tells the father that they have to go to the reception room to do reception procedures (observation). Very good, she makes Jo trust her before she introduced the procedure situation (interview, father). When I meet a child without knowing how the child reacts and how the child is, I will build a relationship, and play and talk to the child before I start with [the] procedure (interview, nurse).	Getting to know each other before introducing the procedure Getting the child to trust the nurse before the procedure starts Building a relationship with the child	Treatment-centered care	Building relationships – gaining trust
Kim wakes up and is about to have the breakfast. The mother tells the nurse that Kim has a poor appetite and eats sparsely. The nurse asks the child directly what the child wants to eat. Afterwards, the nurse brings the child the food the child wants. The mother and the nurse help the child to sit upright in bed. When the nurse returns to the room, the food is almost untouched. The nurse asks what to order for dinner. The nurse has given the child fever-reducing medication before Kim’s favorite dish is served. Kim takes only a few bites (observation). Kim got something else to eat than what was on the menu. The nurse said she would do her best to get something that the child likes. However, Kim would not eat. The child has no appetite. My responsibility is to be there as a mum; take care of my child, do things Kim wants, give Kim something to drink and such (interview, mother). The mother’s task is to be there for the child and offer the child any drinks the child wants. My task is to monitor the child’s physical condition (interview, nurse).	The nurse provided desired food because of poor appetite Personalizing and facilitating the meal for the child in collaboration with the mother Providing desired food related to poor appetite Ensuring that the child receives food and drink Taking care of the child’s physical condition	Home-like care	Making the meal familiar

## Results

The two essential themes with subthemes that emerged from the analysis, illustrating two dissimilar care situations with different purposes, make up the structure of the findings below.

### Treatment-centered care

The aim of treatment-centered care was to perform diagnostic procedures and to carry out treatment of the child in their best interest; this type of care was related to the cause of the child’s hospitalization. We found that parents and nurses collaborated by sharing responsibility and tasks in a dynamic way. The starting point for the nurse was building a relationship and achieving the child’s trust before then enlisting the child’s voluntariness. The nurse and parents further distracted and comforted the child to make him/her feel safe. To ensure the child’s voluntariness and to carry out the procedure with as little resistance and protest as possible, collaboration was essential. In the worst-case scenario, the need to use force caused the child discomfort, and this could increase procedure time. In some situations, the nurses would sideline the parents and call for other nurses to support them. In the treatment-centered care, the initiators were the nurses: they had the main responsibility of carrying out the procedures and treatments and of delegating tasks to the parents. When performing procedures, nurses and parents balanced their actions in a flowing, collaborative way to safeguard the child’s well-being.

The collaboration visualized below in the written narrative of Jo (a fictitious common unisex name) embodies the most common and ideal collaborative situations as observed and expressed by participants. The story (quotations in italics, authors’ comments in bold italics) is organized in accordance with the subthemes.

### The narrative of Jo

#### Building relationships – gaining trust

Jo, who is a preschool child, has the fifth scheduled hospitalization; doctors are searching for a diagnosis. The child is with the father. When the nurse meets the child and the father, she first greets Jo and then the father. Next, she involves herself in the child’s play with a train. She does this before introducing the child to the procedural situation. The father said the following about the nurse’s way of building a relationship with the child: *Very good, she makes Jo trust her.* The nurse confirms this: *When I meet a child without knowing how the child reacts and how he/she is, I will build a relationship, play with and talk to the child.*

***The starting point for the nurses involved building a relationship with the child by playing together in order to achieve the child’s trust. This was done before they introduced the child to the procedural situation. The parents’ role here was to support the nurses.***

#### Securing – gaining voluntariness

After a while, Jo has to undergo different procedures. The nurse brings the equipment to measure oxygen and Jo’s pulse. She shows how it is done on her own hand, and then she wants to do it on the child. However, the father tells her to perform the procedure on him first. Afterwards, he lets the child play with the finger equipment. The father puts it on the child’s finger, and the nurse makes it exciting for the child by showing Jo the rhythm on the screen. The nurse then wants to measure the child’s blood pressure, but the father asks if it would be better to wait until the end of this procedure.

The nurse and the father collaborate when weighing the child and measuring the child’s height. They explain gradually what will happen. The child is on the scale, and the nurse and father stand on either side of the scale in front of the child. The nurse says the child’s weight aloud and boasts about how big the child is. Afterwards they measure the child’s height together. In the interview, the nurse explains the situation: *I show the procedure on myself. However, not everything can be shown; one must try to render it harmless and show what we will do next.* The father says: *I am trying to keep the disagreeable thing at the end. It was done in this way, and Jo was not forced into things that he/she did not like.*

***In this way, the nurses together with the parents prepared the child to perform the required procedure. The father was active, engaged, and worked ahead of what was going to happen to the child. He often took the initiative. The nurse followed the father’s advice. Therefore, they limited the need to force the child and instead enlisted the child’s voluntariness. The parents’ deep knowledge of the child allowed them to become involved in these situations with different but collaborative contributions.***

#### Distracting and comforting, and securing and gaining voluntariness

Upon seeing the blood pressure cuff in the nurse’s hand, the child starts screaming. The father then asks the nurse if she can measure his blood pressure first. She wraps the cuff around the father’s wrist and begins to measure. The nurse then addresses the child, who is in his father’s lap, and together with the father, they try to place the cuff around the child’s arm, but the child screams more intensely and turns away. The father says it does not hurt; it just feels a bit tight. The nurse decides it is best to wait, stops the procedure and says: *Everything went well until we pulled out the cuff and the child screamed, wriggled and turned away. Then it was best to postpone it, because if you proceed, then the relationship you have tried to build deteriorates.*

***The nurse emphasized the child’s discomfort, and her choice to postpone the procedure. This way she maintained her relationship with the child; that was her overriding aim. The father had the overview of the situation. Based on his knowledge of his child, he supported the child, but the nurse made the decision to end the procedure.***

Later in the day, the nurse says to the father that she would like to take a blood pressure measurement before administering the narcosis. As soon as the nurse with the equipment enters the dining room where they sit, the child starts to cry. The nurse says aloud that one should not do unpleasant things in this room, but that this was appropriate right now. The father quickly moves the child from the couch to the computer. The nurse and the father stand behind the child and bend over the child. The nurse sits down next to the table and plays with some small animals with the child on the computer table. The nurse shields the child’s view of the blood pressure measurement equipment, and the child forgets about it after a while and stops crying. The nurse says the animals’ names or alternates between doing this and asking the child about their names. They place the animals together in rows. The nurse asks about the sounds the animals make, and they make the animals’ sounds together. She maintains the relationship by playing with the child, and the father is participating in this play.

The nurse then stands, brings out the blood pressure cuff, and together with the father wraps the cuff around the child’s arm. The child protests and cries. They tell the child that it is not dangerous. At the same time, the father finds pictures of trains on the computer. He shows the trains to the child. The nurse, child and father look at the pictures together. The child’s cry calms down, and the nurse measures the blood pressure. When the cuff is tightened around the arm, the child cries more. The father continues to show new pictures, but the child start crying again loudly when the cuff is tightened on the arm. The father comforts and calms the child. The nurse ends the situation by talking about and playing with the animals with the child, just as they did before the measurement. The nurse says the following about the father: *He was good at distracting and smart to say that it is not dangerous. He stayed with the child, took care of the child, and comforted the child.* The father says: *It may be my task as a parent to distract, comfort, maybe inspire, in order to change the focus and to forget about what is disagreeable.*

***The phases of distracting and comforting the child lasted a long time, and moved back and forth. In this way, the adults avoided or reduced the need to force the child into submitting to the procedure that worried the child. Nurses and parents collaborated in a dynamic way by distracting and comforting the child to safeguard the child and enlisted the child’s voluntariness with the common aim of performing the procedures and taking care of the child’s well-being. The father and nurse were able to establish a physical distance from the equipment by using the computer as a toy and by playing with the toy animals. Both the computer and the animals became tools for distracting and rendering the situation harmless to the child. The father’s deep affiliation with and knowledge of his child enabled him to take the initiative in some of the situations. The nurse inspired the father and also allowed him space to act. In a dynamic way, based on reciprocity, they complemented each other.***

***However, in some situations we observed that the responsible nurse might include other nurses for support, a move which sidelined the parents. This happened when the parents became evasive, which made the child feel insecure. In such situations, the focus was mostly on performing the procedure with less attention on the child.***

### Home-like care

The aim of home-like care is to safeguard the child’s everyday situations in an unfamiliar and strange environment. Maintaining familiar routines were prioritised by the nurses and parents, with varying degrees of collaboration, with the aim of individualizing situations by making familiar meals, maintaining normal sleep patterns, adjusting washing and getting dressed in new situations and normalizing the time in between various situations. The parents were the initiators and were mainly responsible for asking for assistance. The nurses shared responsibility and tasks with the parents, but their involvement with the child was more indirect. Sometimes, the nurses might take over some tasks based on the assessment of needs, and at other times the nurses kept their distance because it was in the best interest of the child.

The child’s illness, the child’s severity, the age of the child, the parents’ previous hospital experience and their presence in the hospital were conditions that gave rise to variations in the degree of nursing involvement. The narrative of Kim (a fictitious common unisex name) embodies the findings of the most ideal and common collaboration from observations and interviews. The story (quotations in italics, authors’ comments in bold italics) is organized in accordance with the subthemes.

### The narrative of Kim

The preschool child Kim is bedridden with a high fever due to a urinary tract infection making the child very ill. The mother is with her child, and they share a room with another family.

#### Maintaining normal sleeping patterns

Kim has been sleeping restlessly during the night due to fever and discomfort. The nurse reports on this and looks into the room in the morning. The light in the room is dim, and the beds are close to each other. The mother lies half in her own bed, but she rests her head in Kim’s bed; a screen surrounds them. The nurse whispers to the mother, and they agree to let Kim sleep. The nurse says that she should not disturb them, and she invites the other family to eat in the dining room. She encourages them to stay in the playroom after breakfast.

***In this way, nurses and parents collaborated to maintain the child’s normal sleeping patterns and enable the rest and security of both the mother and child. The mother was responsible in this concrete situation: she stayed physically close to her child and made a home-like shelter for herself and her child.***

#### Making the meal familiar

After a while, Kim wakes up and is about to have the breakfast. Kim has a poor appetite and eats sparsely. The nurse asks the child what he/she wants to eat. The nurse brings the child the food he/she wants, and the mother and the nurse help the child to sit upright in bed. When the nurse returns to the room, the food is almost untouched. The nurse asks what she should order for dinner. The nurse has given the child fever-reducing medication before dinner – which is Kim’s favourite dish – is served. Kim takes only a few bites. The mother says in the interview; *Kim got something else to eat than what was on the menu. The nurse said she would do her best to get something that the child likes. However, Kim would not eat. The child has no appetite. My responsibility is to be there as a mum: take care of my child, do things Kim wants, give Kim something to drink and such.* The nurse confirms the mother’s role: *The mother’s task is to be there for the child and offer her any drinks the child wants. My task is to monitor the child’s physical condition.*

The next day the nurse also serves breakfast. After a while she returns to the room; the mother and the child are eating at the table. The nurse comments on how much better the child looks and how cosy they are. She leaves the room after a short time.

***Both situations show that the parent and nurse had the common goal of getting the child to eat. Making the meal familiar to the child was the parent’s responsibility. The nurse made practical facilitations to make the meals attractive and offered support by supplying the necessary medication and being flexible with the meals.***

#### Adjusting washing and getting dressed in a new situation

After breakfast the first day, Kim has been washed while lying in bed and has the shirt changed with the mother’s help. The nurse supports them with necessary equipment. The child is still affected by fever. The next day, the child has slept well during the night, and he/she feels better. Kim is dressed before the nurse enters the room.

***In the first situation, the mother needed the nurse to facilitate collecting equipment; however, the next day, she did it alone. In this way, nurses collaborated with parents and adjusted the process of washing and getting dressed to the child’s new situation so that the parents could perform the activities.***

#### Normalizing the time in between

The next day, the child is better, and the nurse informs the mother and child about an activity room where the child can play with a teacher. The child does not initially show any interest, but after a while, the mother follows the child in. Later in the day, the nurse asks if Kim has been in the activity room; the mother responds yes, and the child nods. The mother points to what Kim has made – a bird hanging on the bed – and the child shows it off proudly. The nurse brags and says she will go with the child the next day and make a bird. The mother says: *Doing activities with my child – I will say that is my responsibility. I promised to be with Kim for activities and sit there as long as Kim wanted. Kim feels better when he/she gets to do things and not just sit in the room.* The nurse says: *Taking the initiative in relation to the child is my job as well as collaborating with the mother.*

***Parents and nurses varied their degree of collaboration when maintaining routines well known by the child in everyday situations depending on the level of the child’s illness and treatment. However, the parents had the main responsibility. In some situations, when the child’s illness and treatment presented challenges to everyday situations, the nurses might take over and perform treatment-care to safeguard the child’s well-being. Based on the complexities of the care situation, parents and nurses might change their roles and responsibilities. The degree of the nurses’ involvement varied from weak to moderate to high in order to attend to the child’s everyday needs.***

***We observed that these areas of collaboration depended upon the nurse’s sensitivity to the child’s reactions, as well as input from the parents. In addition, the parents’ input relied on good interactions between parent and child and was based on the parents’ knowledge and affiliation with their child. In this way, the collaboration between nurses and parents was characterized by flexibility and reciprocity and was based on dialogues in action.***

## Discussion

The aim of this study was to explore the experiences of parents and nurses and the concrete ways in which nurses and parents collaborate in partnership when caring for hospitalized preschool children. The findings revealed two characteristics of ideal collaboration between nurses and parents in this context: flexibility and sharing of responsibility and tasks. The findings suggest that there are distinct areas of responsibility for nurses and parents and distinct purposes for the care work performed. The nurses and parents in this study took turns taking the initiative and supporting each other’s goals and actions in partnership. While the parents were responsible for maintaining home-like care, the nurses assumed the primary responsibility for treatment-centered care. Both nurses and parents were dependent on each other’s help to sustain this responsibility, but the relationship changed in accordance with the level of severity of the child’s illness. Flexibility within both areas of collaboration depended on the nurse being sensitive to the child’s needs and taking the parents’ input into consideration. Furthermore, effective interaction between the parents and children, based on the parents’ knowledge of and affiliation with the child, was also a precondition for ideal collaboration. These issues are discussed in more depth below.

### Common and dissimilar goals and roles

In line with international research, the nurses took the initiative and had the responsibility of organizing and performing procedures and treatment [7–13]. In our study, we observed that the collaboration between nurses and parents was dynamic with the aim of ensuring the child’s willingness to perform the procedures. The study further emphasises that the parents’ presence and active input was a necessary factor in making the child feel secure and a precondition for ensuring the child’s voluntariness. The nurse and parents shared the unspoken common goal of performing the treatment because it was necessary.

The parents’ input was based on the parents’ knowledge of and strong attachment to the child. This made the parents secure in their role; therefore, they were able to participate in an active manner. This contributed to the child’s willingness to be treated with the use of as little force as possible. Having the child’s best interest as a common goal allowed a flow of mutual collaboration and dialogue between nurses and parents. In several situations the nurse’s initiative and their playing with a child was important both to establish trust between nurse and child and to distract and comfort the child. The nurse had the main responsibility of carrying out the treatment, which is in line with earlier studies.

Parents performed home-like care, and they regarded this as their responsibility. This is also referred to as daily basic care in international studies [7, 9, 10, 13–19]. Our study provides supplementary findings: for example, parents took care of the child affected by the disease or treatment and maintained the child’s rhythm of everyday situations at home in an unfamiliar environment in order to make them more familiar. To do this, nurses and parents varied their degrees of collaboration when individualizing the child’s situations. In some situations, the nurses had to take over and perform treatment-centered care.

We argue that the nurse’s knowledge and contextual experience-based sensitivity to the child’s reactions and the parents’ contributions enabled the flexibility in both areas of collaboration. This is in line with current theory, which includes parent-professional collaboration in family-centered care and partnership-in-care. The emphasis is on the importance of supporting parents in their role, valuing parents’ knowledge and experience, and incorporating parents’ expertise in developing effective parent-professional relationships as collaborative processes [2]. Parent-professional collaboration and partnership in care, as part of family-centered care, aligns with Norwegian legislation that regulates the rights of parents and hospitalized children: parents have the right to stay together with their child, and to not lose their income when staying at hospital [27]. This right is supported by the right of public paid health care [28].

In this triangular relationship between parents, nurse and child, the child was the primary receiver of care, and both parents and nurses were caregivers in a mutual, dynamic and dialogic collaboration. At the same time nurses and parents switched roles as caregivers and care receivers in relation to each other; they needed each other’s help. This may be described as a collaborative hierarchy, where the participants switched places and roles based on the care situations.

### Contextual sensitivity and reciprocity - make the care safe

The nurse’s professional knowledge and procedural skills, knowledge of how to interpret the child’s and the parents’ reactions, as well as their ability to enter into play with the child seem to be preconditions for correct decisions and actions in a complex collaborative situation. The division of responsibility, characterized by reciprocity based on dialogue in the collaboration, happened in accordance with what Tove Pettersen defines as mature care. Pettersen claims that in situations where it is necessary to change perspective and assess possibilities and limitations in order to find solutions, being able to interpret the care receiver’s expression is a precondition. This requires that one possesses contextual sensitivity in the situation [29].

The collaboration was also in accordance with Pettersen’s [30] argument that care receivers are not passive receivers but rather active in the relationships and thereby equal participants. In mature care, the care is administered in dialogue with the receiver, that is, in a partnership, and it is done in a dynamic way where empathy with the care receiver is of significance. This is in accordance with the intention of the Convention on the Rights of the Child [31]. Parents represented the child based on knowledge and the child’s attachment in accordance with the theory of attachment [32]. This contributed to balancing the use of force on the child in procedural situations and to furthering the child’s willingness to submit to procedures.

### The limits of care

When performing the ideal care, nurse and parents had an unspoken, common goal of carrying out the treatment because it was necessary. They worked in a dialogical relationship to achieve this. The child’s emotional attachment to the parents challenged the parents to provide unambiguous input in the collaboration as a response to the child’s need. This is, however, in contrast to situations where the parents or nurse become insecure in their role and evasive in the collaboration, and the child becomes insecure and less willing to submit to treatment. The procedure time may then increase, or it may become necessary to postpone the procedure. This presupposes a context that allows for time to develop a partnership and to establish a feeling of calm in procedural situations.

When parents change the way they react to the child’s needs, the circle of security is broken and the children become insecure. The child’s circle of security is broken because the parent’s role changes [33]. This is in line with John Bowlby’s theory of emotional attachment between children and parents based on continuity in the parents’ response to the child’s needs. Where the attachment contact between parents and children were good i.e. physical contact such as the physical embrace of the child, the collaboration was successful. This may also happen if the nurse takes over treatment-centered care in home-like care situations. This may be in opposition to what Pettersen [29] points out: Mature care between caregivers and care receivers, according to the principle of reciprocity, maintains a balanced use of power, where dialogue is central. This is a challenge to the dialogue and the reciprocal relationship. We observed that where the circle of security is disturbed, the nurses needed to take on more responsibility and include the parents in the situation, as well as fulfil their responsibility of carrying out procedures and making the child feel safe. Assuming the responsibility for parents and child at the same time is demanding, and requires that the nurses have matured with regard to both professional knowledge and skills.

Despite feeling empathic for the child and the child’s lack of willingness to receive treatment, the nurses did not refrain from performing procedures and administering treatment. They may have sidelined the parents by calling in other nurses to assist if necessary. Parents were then bumped down in the collaborative hierarchy, so that the nurse was able to perform the necessary assessment and treatment of the child. Thus, the nurse’s relationship with the parents and especially regarding treating and diagnosing the child experienced some challenges. According to Pettersen these are the situations where the limit of care for the nurse has been reached. A way of limiting the use of force was to limit time spent in situations that caused the child discomfort. In order to carry out procedures/treatment, the nurse included new nurses in the situation to help, but this was not done until other approaches had been tried out.

The partnership role between parents and nurses is in line with the notion of parent-professional collaboration in family-centered care [2]. Nurses and parents assume distinct areas of responsibility, distinct purposes for the care work, and distinct roles, as affirmed by international literature [8]. Despite these differences, understanding of the partnership between nurses and parents can serve a bridge-building function to connect the differences and create a common goal for the best interests of the child.

## Strengths and limitations of the study

The study was conducted in one small general medical paediatric unit of a Norwegian hospital with children with different medical diagnoses. These findings could be of value in similar contexts and cultures but not in relation to children admitted to intensive care units. The intention was to obtain in-depth knowledge of parents’ and nurses’ experiences. Possible weaknesses may be that the study was performed in one hospital in one geographical area, and that the findings are related to a specific culture. The study’s strengths lie in the method used: a field study, combining participant observations and qualitative interviews. The visual access to the process reinforces the possibility of following up on core aspects of the care situations by carrying out in-depth interviews. These would provide deeper insights into the area of research.

## Conclusion

The aim of the study was to explore parents’ and nurses’ concrete collaborative experiences. The findings describe two ways of collaborating in the best interest of the child. Collaboration in treatment-centered and home-like care has different purposes and is linked to different situations even though the situations may interfere with each other. Moreover, collaboration is based on the parents and nurses having different responsibilities. In order to safeguard the child’s best interest, collaboration between nurses and parents was characterized by flexibility and reciprocity and by dialogues in action. Areas of collaboration were characterized by the nurse’s sensitivity to the child’s reactions as well as by input from the parents. Parents depended on good interactions with their children based on their knowledge of and affiliation with the child. The findings showed that parents and nurses’ partnerships were central to describing the ideal collaboration between them.

## Relevance to clinical practice

The findings may be of use to families with children admitted to general children’s wards. In terms of clinical practice, the findings may present nurses with the possibility of collaborating flexibly and in partnership with parents. These perspectives of the combination of the differences and the partnership is important in the nurses understanding of their roles and as a fundamental part of their practice. It is therefore necessary to include and emphasize the perspectives in the education of nurses. Ways to learn about the nursing role for the students are in practice, in simulation of relevant cases and with reflection in practice over the nursing role. The leadership of the nursing’s practice have to facilitate this fundamental perspective of the nurse’s role and as a basis for the nursing practice.

## Data Availability

The data analyzed during the current study are not publicly available due to an agreement with the participants on the confidentiality of the data, but are available from the corresponding author on reasonable request.
